# Strategic Combination of Isocratic and Gradient Elution for Simultaneous Separation of Polar Compounds in Traditional Chinese Medicines by HPLC

**DOI:** 10.1155/2018/7569283

**Published:** 2018-03-19

**Authors:** Mei-Tuo Zhang, Xiao-Xue Ye, Wei Lan, Yan-Ling Yang, Tsunghsueh Wu, Yu-Sang Li, He-Bin Tang

**Affiliations:** ^1^Department of Pharmacology, School of Pharmaceutical Sciences, South-Central University for Nationalities, Wuhan, Hubei 430074, China; ^2^Key Laboratory of Analytical Chemistry of the State Ethnic Affairs Commission, College of Chemistry and Materials Science, South-Central University for Nationalities, Wuhan, Hubei 430074, China; ^3^Department of Chemistry, University of Wisconsin-Platteville, Platteville, WI 53818, USA; ^4^Research Institute of Huazhong University of Science and Technology in Shenzhen, Shenzhen 518057, China

## Abstract

A simple high-performance liquid chromatography (HPLC) method for the simultaneous separation of the highly polar and weakly polar components of traditional Chinese medicines was developed via a strategic combination of isocratic and gradient elution methods. Liu-Shen-Wan and Liu-Wei-Di-Huang-Wan were used as representative examples of traditional Chinese medicines. This is the first time that 6 components of varying degrees of polarity in Liu-Shen-Wan had been successfully resolved in a single chromatographic run using an ultraviolet-visible detector with a fixed wavelength of 296 nm. In contrast to conventional gradient separation methods, this novel method offered a viable route for separation of the highly and weakly polar fractions simultaneously, thus greatly reducing the time and cost of analysis. This method therefore provides a more efficient way to determine the polar components present in traditional Chinese medicines. It would find potential application in drug screening, drug authentication, and product quality control.

## 1. Introduction

In contrast to western medicines, which are normally produced by chemical synthesis, traditional Chinese medicines (TCMs) are composed of herbal ingredients that exhibit certain biological and/or pharmacological properties. However, the bioactive constituents present in TCMs and the mechanisms of action are poorly understood, although it is widely accepted that the synergic effect of various ingredients is responsible for the therapeutic effects of TCMs [1, 2]. Currently, a number of systematic studies have focused on identification of the chemical components in TCMs, their pharmaceutical activities, and quality control, with the overall aim of gaining an improved understanding of TCMs and thus ultimately lead to their widespread usage [3]. Indeed, the quality control of herbal treatments has remained a bottleneck, hindering their development due to the multiple chemical constituents present in these complex systems [4]. Hence, effective analytical methods are required to ensure safe and efficient application of herbal preparations.

Liu-Shen-Wan (LSW) is a traditional TCM commonly used for the treatment of localized infections and inflammation and the alleviation of associated pain [5]. Indeed, this ancient medicine has exhibited considerable efficacy in treating a number of illnesses, such as diphtheria, scarlet fever, pharyngotonsillitis, acute tonsillitis, purulent parotitis, encephalitis B, and viral pneumonia [6]. According to previous studies, the major chemical components present in LSW are Realgar, Bufonis venenum, Moschus, Calculus bovis, Margarita, and Camphora [7]. Another common TCM is Liu-Wei-Di-Huang-Wan (LWDHW), which contains prepared Rehmannia root, Corni fructus, Dioscoreae rhizoma, Alismatis rhizoma, Moutan cortex, and Poria and acts to nourish the Yin, tones the kidneys, balances the Yin and Yang, enhances physical strength, and regulates the immune system [8–11].

In general pharmaceutical practice, analytical fractions containing weakly polar molecules are commonly separated and studied in great detail, while fractions containing molecules of high polarity are often ignored. Due to this bottleneck, there is a lack of understanding of the therapeutic effects of the highly polar compounds present in TCMs, and even the synergic effects originating from these larger constituents may be overlooked. Owing to the close relationship between TCM and Chinese classical philosophy, the effect of TCMs on balancing the Yin and Yang is of particular interest. Therefore, an efficient HPLC method to effectively analyze all constituents present in TCMs such as LSW and LWDHW is required.

Among the various methods reported for the separation of highly polar compounds, ion-pair liquid chromatography and hydrophilic interaction liquid chromatography (HILIC) have been widely accepted despite their high costs [12, 13]. However, these methods are limited to the simultaneous qualitative or quantitative analysis of specific components and thus cannot accurately account for all polar fractions present in TCMs.

Thus, we herein report our studies into the development of a rapid, low-cost, and extensive combinative elution method involving a strategic combination of isocratic and gradient separation on a C_18_ column to achieve the highly efficient separation and identification of the chemical constituents present in ethanolic and aqueous liquid extracts of LSW and LWDHW.

## 2. Materials and Methods

### 2.1. Reagents and Materials

The 5-hydroxytryptamine standard was obtained from the National Administration of Food and Drug Control of China; bufotalin, cinobufotalin, bufalin, cinobufagin, and resibufogenin standards were purchased from Shanghai Yuanye Bio-Technology Co., Ltd. (China); LSW was obtained from Shanghai Leiyunshang Pharmaceutical Co., Ltd. (China); and LWDHW was purchased from Beijing Tongrentang Pharmaceutical Co., Ltd. (China). In addition, HPLC grade methanol and acetonitrile were obtained from Tedia Co., Inc. (USA). Ultrapure water (18.2 MΩ·cm) was employed in all experiments and was obtained from a Milli-Q® apparatus (Millipore, Bedford, MA, USA).

### 2.2. Instrumentation and HPLC Parameters

HPLC and LC analyses were carried out using a Dionex™ Ultimate 3000 system (Thermo Fischer Scientific, Inc., USA) equipped with an Ultimate 3000 pump, an Ultimate 3000 autosampler, and an Ultimate 3000 column compartment. For HPLC, an Ultimate 3000 diode array detector was employed, and for LC, an Ultimate 3000 variable wavelength detector was employed.

Separation was performed using a C_18_ column (4.6 × 250 mm, 5 *µ*m, Dionex) and an optimized mobile phase composed of acetonitrile (solvent A) and 0.1% trifluoroacetic acid water solution (solvent B, v/v). For a conventional separation, the column was eluted using a linear gradient of 90–50% A for 25 min, 50% A for 35 min, and then 90% A for the final 10 min (total run time 45 min). For the combinative elution, elution was achieved using the following conditions: (i) isocratic elution with 5% A from the injection time until 3 min, (ii) a gradient from 5% A to 12% A over the next 10 min, (iii) a further linear gradient from 12% to 20% A for 2 min, (iv) 30% A over 10 min, (v) 51% A for 5 min, and finally (vi) isocratic elution with 51% A for 20 min (total run time 50 min). The detection wavelength was set at 296 nm, and the flow rate was 1.0 mL/min. The column temperature was set at 30°C, and the injection volume was 10 *µ*L.

### 2.3. Sample and Standard Preparation

#### 2.3.1. Standard Preparation

A stock solution was prepared by dissolving a sample of the standard (5-hydroxytryptamine; 10.00 mg) in ultrapure water (10.00 mL) in a volumetric flask to give a concentration of 1.000 mg/mL 5-hydroxytryptamine. Working solutions were then prepared by serial dilution of the stock solution in volumetric flasks to give solutions of the following concentrations: 0.33, 0.11, 0.037, 0.012, 0.004, and 0.001 mg/mL. The various stock and standard solutions were either prepared freshly prior to use or were stored at 4°C following preparation until required. Before use, all samples were filtered using a 0.45 *µ*m syringe filter.

#### 2.3.2. Preparation of the Water-Soluble Pharmaceutical Samples

LSW or LWDHW pills were milled to produce a powder, and a sample of this powder (91.00 mg) was mixed with ultrapure water (5 mL) using ultrasonication at 50 kHz for 30 min. Any undissolved solids were allowed to settle to allow facile transfer of the supernatant. After removal of the supernatant, the remaining solids were extracted once again using ultrapure water (5 mL), and the supernatant samples were combined. The extracted constituents were obtained as a yellow powder following freeze-drying. The sample solution for analysis was then prepared by redissolving a sample of the yellow powder (6.60 mg) in water (5 mL) in a 5 mL volumetric flask. Prior to use, the sample solution was filtered using a 0.45 *μ*m syringe filter.

#### 2.3.3. Preparation of the Ethanol-Soluble Pharmaceutical Samples

The method employed for preparation of the ethanol-soluble pharmaceutical samples was as described above for the water-soluble samples, with the exception that ethanol was used at all stages rather than ultrapure water. In this case, the extracted constituents were obtained as a brown powder following freeze-drying.

## 3. Results and Discussion

### 3.1. Comparison of Two Different Separation Modes

**Figure 1:**
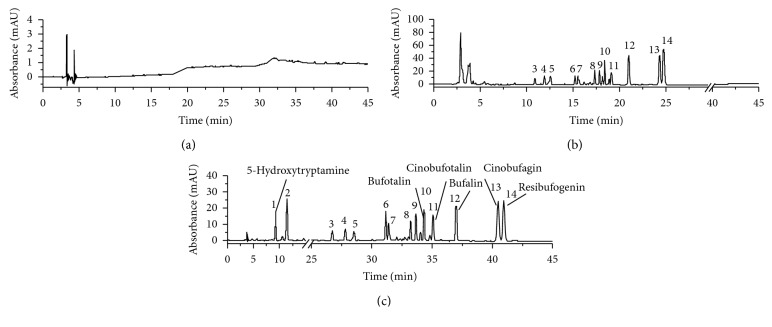
Separation of the alcohol-soluble extractants of LSW by reverse-phase HPLC using (a) a combinative elution method and (b) a conventional gradient elution method. (c) The blank. The peaks 1, 10, 11, 12, 13, and 14 correspond to 5-hydroxytryptamine, bufotalin, cinobufotalin, bufalin, cinobufagin, and resibufogenin, respectively.

In the analysis of traditional Chinese medicine formulations, gradient elution methods have commonly been employed due to difficulties in separating the complex mixture of components present in TCMs. Indeed, gradient separation works well for many TCMs, and it represents the simplest and most convenient form of liquid chromatography. However, the efficient separation of highly polar components is a challenge due to their poor retention in such systems. As shown in Figure 1(b), no single mode can provide satisfactory separation of the highly polar compounds present in LSW (such as 5-hydroxytryptamine, peak 1) by conventional reverse-phase HPLC. Due to their rapid elution from the system, the peaks corresponding to the most polar analytes were completely overlapped with the solvent peak. However, upon the application of an elution gradient followed by a 3 min isocratic elution, two highly polar compounds (represented by peaks 1 and 2) were clearly separated from the solvent peak (Figure 1(a)), indicating that gradient elution was successful in increasing the retention times of these compounds.

We therefore carried out careful optimization of the HPLC system to develop the proposed separation method. During this optimization, we considered that a suitable compromise between resolution and analysis time should be reached, and so the mobile phase composition and elution procedures were examined in detail. In the preliminary experiments, various mobile phases based on acetonitrile and water and containing trifluoroacetic, formic, and phosphoric acid were tested. Although acetonitrile and water elution have commonly been employed for the separations of weakly polar molecules in TCMs, the elution performs poorly in the separation of highly polar molecules. We also found that the addition of trifluoroacetic acid provided better results than formic or phosphoric acid [6, 14]. Indeed, the optimal separation of the 14 components of interest was achieved through a strategic combination of isocratic and gradient elution stages using acetonitrile and a 0.1% (v/v) aqueous solution of trifluoroacetic acid over 50 min. Other parameters such as flow rate, injection volume, and column temperature were optimized to stabilize the retention times.

### 3.2. Effect of Solvent for the Extractable Constituents

Recently, the anticancer properties of LSW have been studied and reported, thus resulting in increased research efforts to identify the highly sought-after active pharmaceutical ingredients [15]. In our previous studies into the effects of sample pretreatment, constituents extracted from LSW using ethanol exhibited enhanced therapeutic performances and potencies compared to the constituents extracted using water [16]. Furthermore, the ethanol-soluble components exhibited low toxicity, evidently revealing the ability of ethanol to separate potentially harmful water soluble and highly polar components from the LSW matrix. However, to date, the underlying mechanisms for the beneficial and toxic effects arising from the two groups of extracted components have yet to be determined [16–18]. Ironically, hot water extraction has traditionally been the most common route to generate pharmaceutical doses of TCMs for Chinese patients. Consequently, HPLC analysis of the various constituents obtained via ethanol and water extraction is of particular interest in the context of pharmacochemistry.

**Figure 2:**
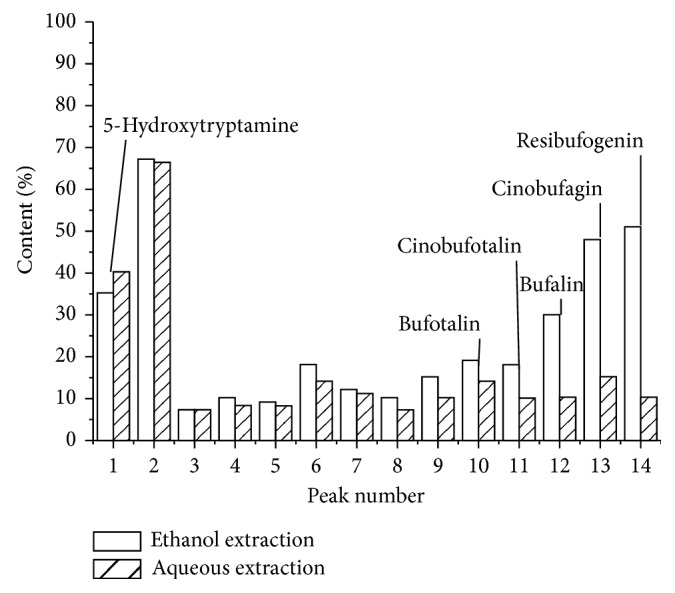
Comparison of the relative contents of the 14 LSW components in the ethanolic and aqueous extraction liquors of LSW. The peaks 1, 10, 11, 12, 13, and 14 correspond to 5-hydroxytryptamine, bufotalin, cinobufotalin, bufalin, cinobufagin, and resibufogenin, respectively.

Thus, we herein prepared a solution of LSW as described in Section 2, and the 14 constituents present in the alcoholic and aqueous extractants of LSW solution were determined using the optimized HPLC conditions. A quantitative analysis of the 14 components was then performed using 5-hydroxytryptamine as an internal standard, and the results are summarized in Figure 2. As shown, similar contents were observed for the aqueous and ethanolic extractions of components 1, 2, 3, 4, 5, and 7, while the contents of components 6, 8, 9, 10, 11, 12, 13, and 14 varied to a greater extent, with peaks 12, 13, and 14 (which corresponded to bufalin, cinobufagin, and resibufogenin, resp.) exhibiting threefold enhanced alcohol solubility compared to the content found in their aqueous extractions. These results indicate that the various components present in LSW exhibited slightly different solubilities in ethanol and water. Such differences are of particular interest because TCM is based on the “Holism” philosophy and not on the “reductionism” philosophy.

### 3.3. Method Validation

#### 3.3.1. Linearity

Linear regression of the data showed that 6 of the 14 components present in LSW, namely, 5-hydroxytryptamine, bufotalin, cinobufotalin, bufalin, cinobufagin, and resibufogenin, presented good linearity with coefficients of determination (*R*^2^) > 0.999. For analysis of standard 5-hydroxytryptamine, bufotalin, cinobufotalin, bufalin, cinobufagin, and resibufogenin, the calibration relationship can be expressed in turn as follows: *Y*=147.7*X*+0.1858(*R*^2^=1.00), *Y*=176.69*X*+0.4679(*R*^2^=0.999), *Y*=136.34*X*+0.3816(*R*^2^=0.999), *Y*=150.63*X* − 0.2965(*R*^2^=1.00), *Y*=160.27*X*+1.2218(*R*^2^=0.999), *Y*=171.73*X*+0.0584(*R*^2^=1.00), where *X* and *Y* represent the peak area and sample concentration, respectively. These results demonstrate that the six components exhibited good calibration relationship for their concentrations. In addition, 5-hydroxytryptamine exhibited a good liner relationship in the range of 0.001∼0.33 mg/mL. Meanwhile, the other five ingredients have a good linear relationship in the same range of 0.0125∼0.1 mg/mL.

#### 3.3.2. Accuracy and Precision

**Figure 3:**
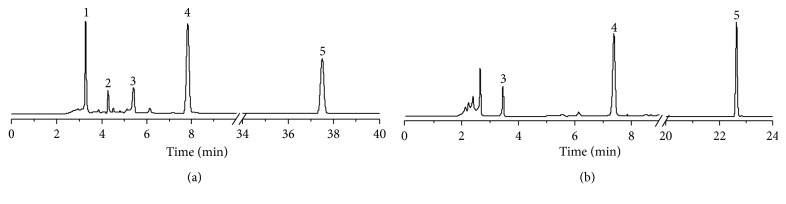
HPLC separation of LWDHW using our optimized combinative elution method (a) and a conventional elution gradient (b).

The accuracy and precision of the method were then evaluated through recovery, repeatability, and stability experiments for the detection of 6 components present in LSW. Detailed recovery and RSD of intra- and interdays for the 6 compounds are shown in Table 1, while the distribution of the mean recoveries is shown in Figure 3. Indeed, the recoveries of the 6 compounds were in the acceptable range of 94.5%–106.9% when the control samples were spiked at the levels of 30, 60, and 90 *µ*g/mL. As represented in Table 2, values reported for spiked samples reflect subtraction of endogenous (no-spiked) value. Therefore, the good recoveries and RSD values obtained herein support the adequacy of the method.

#### 3.3.3. Analytical Limits

The limit of detection (LOD) and limit of quantification (LOQ) of the optimized method were established for each of the 6 compounds of interest outlined in Table 1. As shown, the corresponding LODs ranged from 1.11 to 11.26 ng/mL, and the signal-to-noise ratio was 3. Meanwhile, the LOQ ranged from 3.70 to 37.7 ng/mL with the signal-to-noise ratio of 10. These results are summarized in Table 1.

#### 3.3.4. Application to Other TCMs

LWDHW is also an extremely important TCM, to the extent that its formulation has been patented. However, because of the complexity of its chemical constituents, the pharmacokinetics of this formulation has not yet been clearly elucidated, and as such, its pharmacological properties are not well understood. An efficient method to effectively analyze the various components of LWDHW is therefore required, and so we employed our optimized combinative elution HPLC method to identify the components of LWDHW following its extraction [18]. As shown in Figure 3(a), 5 ultraviolet-active components were obtained, including 2 highly polar components. This contrasts to conventional separation methods, where the peaks corresponding to the most polar analytes overlapped with the solvent peak (Figure 3(b)). These results therefore suggest that our optimized system is suitable for application in the analysis of other TCMs.

## 4. Conclusions

We herein reported the establishment of a simple strategic combination approach (isocratic and gradient elution) for the separation of the various polar components present in two traditional Chinese medicines (TCMs), namely, Liu-Shen-Wan (LSW) and Liu-Wei-Di-Huang-Wan (LWDHW). More specifically, this was achieved through optimization of the high-pressure liquid chromatography (HPLC) conditions to extend the retention times of highly polar fractions, such as 5-hydroxytryptamine, which are generally eluted with the solvent front using conventional gradient separation methods. Indeed, we successfully achieved separation of the highly polar and weakly polar components present in LSW (14 components) and LWDHW (6 components) following their extraction into either aqueous or ethanolic media. The developed method has a number of advantages over hydrophilic interaction liquid chromatography (HILIC) techniques due to its low cost, simplicity, and extensive adaptability. We could therefore conclude that these results may lead to the determination of comprehensive chemical information for further studies into the pharmacology and mechanisms of such TCMs. Furthermore, we expect that our optimized method will be suitable for the analysis of highly polar molecules in other complex TCMs, and it has the potential for application in drug screening and authentication and in product quality control.

## Figures and Tables

**Table 1:** Method validation through the detection of 6 different compounds in the ethanol extraction of LSW.

Peak number	Compound	Intraday RSD (%)	Interday RSD (%)	Recovery (%)	LOD (ng/mL)	LOQ (ng/mL)
1	5-Hydroxytryptamine	0.31	0.38	101.2	8.22	27.4
10	Bufotalin	0.84	1.30	103.2	5.28	17.6
11	Cinobufotalin	0.69	1.42	94.5	7.47	24.9
12	Bufalin	0.13	0.70	106.9	11.26	37.7
13	Cinobufagin	0.16	0.65	104.3	1.11	3.70
14	Resibufogenin	0.15	0.31	101.1	7.81	26.0

**Table 2:** Spike and recovery of 6 components in the ethanol extraction of LSW.

Sample	Low spike (30 *µ*g/mL)	Medium spike (60 *µ*g/mL)	High spike (90 *µ*g/mL)	Mean recovery
5-Hydroxytryptamine	35.34	62.09	86.08	101.2% ± 6.7%
	29.56	60.34	87.29	
	30.27	59.80	87.45	
Bufotalin	28.93	60.53	95.76	103.2% ± 4.8%
	29.46	65.16	96.45	
	29.27	63.61	99.47	
Cinobufotalin	28.88	58.58	83.64	94.5% ± 2.9%
	29.84	55.84	82.15	
	28.65	56.15	81.78	
Bufalin	29.58	61.72	99.41	106.9% ± 3.8%
	32.22	64.59	99.14	
	32.50	64.32	98.30	
Cinobufagin	30.03	65.27	89.03	104.3% ± 4.9%
	32.41	65.79	88.33	
	31.06	65.64	88.82	
Resibufogenin	28.43	64.43	91.77	101.1% ± 5.4%
	28.84	64.49	91.27	
	28.53	64.59	88.16	

## Abbreviations

HILIC: Hydrophilic interaction liquid chromatography
HPLC: High-performance liquid chromatography
LSW: Liu-Shen-Wan
LWDHW: Liu-Wei-Di-Huang-Wan
TCM: Traditional Chinese medicine.
